# Living at the Frontiers of Life: Extremophiles in Chile and Their Potential for Bioremediation

**DOI:** 10.3389/fmicb.2018.02309

**Published:** 2018-10-30

**Authors:** Roberto Orellana, Constanza Macaya, Guillermo Bravo, Flavia Dorochesi, Andrés Cumsille, Ricardo Valencia, Claudia Rojas, Michael Seeger

**Affiliations:** ^1^Laboratorio de Microbiología Molecular y Biotecnología Ambiental, Departamento de Química and Centro de Biotecnología Daniel Alkalay Lowitt, Universidad Técnica Federico Santa María, Valparaíso, Chile; ^2^Departamento de Biología, Facultad de Ciencias Naturales y Exactas, Universidad de Playa Ancha, Valparaíso, Chile

**Keywords:** extremophile, Chile, Atacama Desert, Altiplano, Patagonia, Antarctica, bioremediation

## Abstract

Extremophiles are organisms capable of adjust, survive or thrive in hostile habitats that were previously thought to be adverse or lethal for life. Chile gathers a wide range of extreme environments: salars, geothermal springs, and geysers located at Altiplano and Atacama Desert, salars and cold mountains in Central Chile, and ice fields, cold lakes and fjords, and geothermal sites in Patagonia and Antarctica. The aims of this review are to describe extremophiles that inhabit main extreme biotopes in Chile, and their molecular and physiological capabilities that may be advantageous for bioremediation processes. After briefly describing the main ecological niches of extremophiles along Chilean territory, this review is focused on the microbial diversity and composition of these biotopes microbiomes. Extremophiles have been isolated in diverse zones in Chile that possess extreme conditions such as Altiplano, Atacama Desert, Central Chile, Patagonia, and Antarctica. Interesting extremophiles from Chile with potential biotechnological applications include thermophiles (e.g., *Methanofollis tationis* from Tatio Geyser), acidophiles (e.g., *Acidithiobacillus ferrooxidans, Leptospirillum ferriphilum* from Atacama Desert and Central Chile copper ores), halophiles (e.g., *Shewanella* sp. Asc-3 from Altiplano, *Streptomyces* sp. HKF-8 from Patagonia), alkaliphiles (*Exiguobacterium* sp. SH31 from Altiplano), xerotolerant bacteria (*S. atacamensis* from Atacama Desert), UV- and Gamma-resistant bacteria (*Deinococcus peraridilitoris* from Atacama Desert) and psychrophiles (e.g., *Pseudomonas putida* ATH-43 from Antarctica). The molecular and physiological properties of diverse extremophiles from Chile and their application in bioremediation or waste treatments are further discussed. Interestingly, the remarkable adaptative capabilities of extremophiles convert them into an attractive source of catalysts for bioremediation and industrial processes.

## Introduction

Most the well-described forms of life are mainly adapted to face environments with “physiological conditions,” a term described in literature as moderate temperature (10–37°C), pH ∼ 7, salinity ranging from 0.15 to 0.5 M NaCl, pressure 1 atm and enough water availability (Aguilar et al., 1998; Antranikian et al., 2005). However, there is still a large under-examined group of organisms, known as extremophiles, that are capable of adjust, survive or thrive in hostile habitats that were previously thought to be inhospitable or even lethal for life (Rampelotto, 2013). In general, extremophiles are divided in two categories: extremophiles, which require one or more extreme conditions to grow, and extremotolerant organisms, which can tolerate extreme and/or toxic conditions, although they grow optimally at “physiological” conditions (Canganella and Wiegel, 2011). The study of extremophiles is a rather difficult field, mainly constrained by the complexity of reaching their ecological niches and isolating these microbes. Most extremophiles are still part of the microbial dark matter that has not been discovered yet (Bernard et al., 2018). Hunting microbes in extreme environment is a huge challenge for microbiologists worldwide, which could provide microorganisms, enzymes and biomolecules for diverse applications in biotechnology, biomedicine and industrial processes. The knowledge of the adaptation mechanisms of microbes to extreme environments provides metabolic networks, regulation circuits and pieces for systems biology and synthetic biology (Chen and Jiang, 2018). Extreme conditions drive the evolution of their inhabitants, highlighting the role of extremophiles as models for the study of the evolution of biological entities.

Newly developed technologies have allowed research on extreme environments to gain knowledge on microorganisms and provide significant insights about the origin of life on Earth. Recent studies have uncovered that the first terrestrial life form, known as LUCA (Last Universal Common Ancestor), was a thermophilic anaerobe capable of gaining energy from geochemical sources (Weiss et al., 2016). Basic research has provided valuable insights on how extremophiles can survive such challenging environments. However, the presence of highly sophisticated mechanisms of adaptation together with the availability of a sweet of novel biochemical pathways sustaining peculiar physiological metabolic capabilities converts extremophiles into a current focus of applied research to exploit their biotechnological potential (Rampelotto, 2013). Further attention has also been devoted to identification, isolation and characterization of biomolecules, most of them enzymes named as extremozymes, which are well adapted to be active also at extreme conditions (Rampelotto, 2013).

Extremophiles, can be classified according to the conditions in which they grow: thermophiles and hyperthermophiles (organisms growing at temperatures of 45–80°C and >80°C, respectively) (Madigan et al., 2000; Berenguer, 2011), psychrophiles (organisms that grow at <10°C) (Siddiqui et al., 2013), acidophiles and alkaliphiles (organisms optimally adapted to pH < 5 and pH > 9, respectively), halophiles (organisms that require NaCl for growth, in concentration of 200–5,900 mM) (Edbeib et al., 2016), microorganisms that survive in dry environments (water activity < 0.75) (Connon et al., 2007) and radiotolerant (UV resistant) extremophiles that are resistant to the permanent exposure to damaging solar radiation (Gabani et al., 2014) (Table 1). Additionally, it is worth mentioning that extremophiles are usually defined by one extreme condition, nevertheless, many natural environments possess two or more extreme conditions. The microbiota living on those ecosystems, also known as polyextremophiles, is adapted to an additional extreme condition to the one condition that characterizes them, such as temperature, pH, salinity (Urbieta et al., 2015b).

**Table 1:** General description of extremophiles present in diverse extreme environments of Chile.

Environmental parameter	Extremophile	Definition	Example	Reference
Temperature	Hyperthermophile	Growth > 80°C	*Pyrococcus* sp. M24D13	Dennett and Blamey, 2016
	Thermophile	Growth 45–80°C	*Methanofollis tationis*	Zabel et al., 1984
	Psychrophile	≤10°	*Pseudomonas* sp. ATH-43	Rodriguez-Rojas et al., 2016a
pH	Acidophile	pH < 5	*Acidithiobacillus ferrooxidans*	Demergasso et al., 2010
	Alkaliphile	pH ≥ 9	*Halomonas alkaliphila*	Quiroz et al., 2015
Salinity	Halophile	2,000–5,000 mM NaCl	*Haloferax* sp. CL47	Bonfá et al., 2011
UV radiation	Radioresistant	Radiation tolerant (40–400 nm)	*Deinococcus peraridilitoris*	Rainey et al., 2007
Water availability	Dehydration tolerant	Growth water activity < 0.75	*Streptomyces bulli*	Santhanam et al., 2013


**FIGURE 1:**
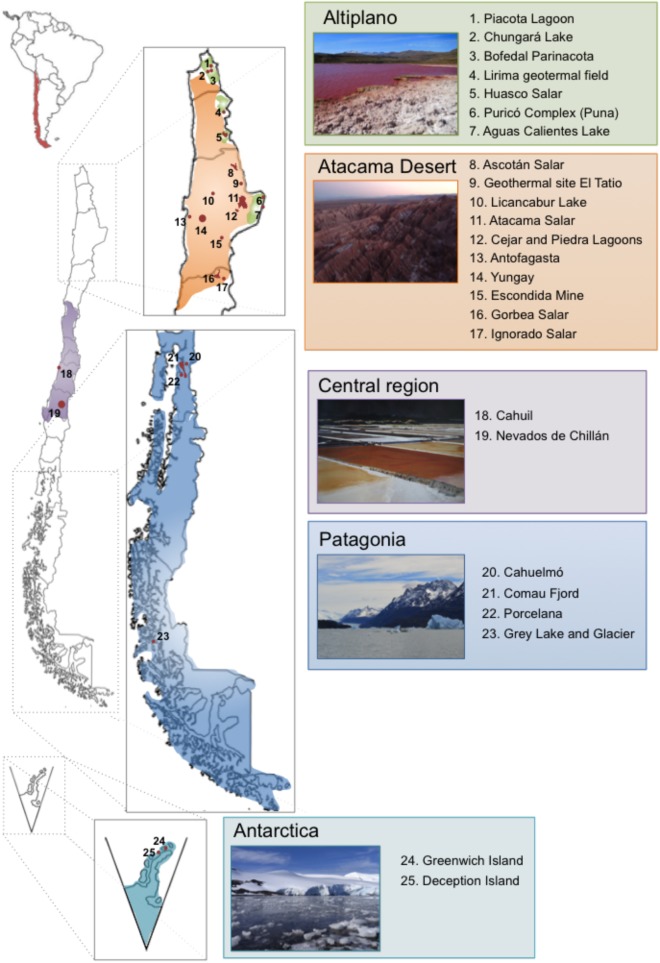
Distribution of different extreme ecosystems harboring extremophiles in five main biotopes throughout Chile. Altiplano (photography: Red Lagoon, Amuyo, Camarones, Parinacota Region), Atacama Desert (photography: Moon Valley, San Pedro de Atacama, Antofagasta Region), Central Region (photography: Cahuil Saltern, Nilahue, O’Higgins Region), Patagonia (photography: Grey Lake and Glacier, Magallanes Region), and Antarctica (photography: Arturo Prat Station, Greenwich Island).

Chile occupies a long strip of territory in the south west of South America and with other countries shared part of the Antarctica. It has been referred to be a “biogeographic island” (Scherson et al., 2017), due to a series of geological events that have formed the current natural barriers Altiplano, Atacama Desert, Los Andes Mountains, Pacific Ocean, Patagonia, and Antarctica (Villagrán and Hinojosa, 1997; Villagrán and Armesto, 2005). Due to its extremely diverse geography and singular geochemical and climatic conditions, Chile gathers many extreme environments, which may result intolerably hostile or even lethal for most life forms, except for extremophiles (Rampelotto, 2013). In order to enable growth under these harsh conditions, extremophiles have been subjected to several adaptations, which have been only partially characterized to date. Altiplano, Atacama Desert, Central Chile, Patagonia, and Antarctica correspond to main geographical areas in Chile that harbor multiple extreme biotopes (Figure 1). Specific extreme biotopes of Chile are attractive scenarios to study the evolution of microorganisms under extreme conditions, which resemble the islands of the Galapagos Archipelago that provide Darwin inspiring ideas to build up the Theory of Evolution by Natural Selection of macroorganisms based on the divergence of species of birds, especially the Galapagos finches.

The preservation of the natural ecosystems and the restauration of polluted sites are crucial for a sustainable development. Bioremediation is an important technology for the clean up of environments contaminated with persistent organic pollutants [e.g., pesticides, polychlorobiphenyls (PCBs), petroleum hydrocarbons] and heavy metals (e.g., Hg, Cd) (Morgante et al., 2010; Saavedra et al., 2010; Rojas et al., 2011; Fuentes et al., 2014; Bravo, 2017). Mining of valuable metals such as copper, gold and silver is historically one the most relevant economic activities in Chile, mainly in the Northern and Central regions. Copper mining is the main activity in Chile, contributing with one third of the world’s production. Unfortunately, this mining activity has been associated with heavy metal pollution in diverse sites. Microorganisms are main biocatalysts for bioremediation of polluted environments and waste treatment processes (Hernández et al., 2008a,b; Saavedra et al., 2010; Rojas et al., 2011; Fuentes et al., 2016; Méndez et al., 2017) and extremophilic microbes are required as catalysts for the bioremediation of polluted extreme environments. The aims of this review are to describe extremophiles that inhabit the main extreme biotopes in Chile, and their molecular and physiological capabilities that may be advantageous for the design and development of bioremediation processes.

## Biotopes and Ecological Niches

Chilean territory is spanned within a long altitudinal and latitude range that contributes to the origin of a suite of landscapes and geological features that are extreme habitats for microorganisms. Some of these ecosystems will be described briefly.

### Atacama Desert

The Atacama Desert is located along the western border of South America and is the driest and oldest desert on Earth’s, exhibiting similar conditions described for Mars. This ecosystem provides a unique collection of habitats, ideal for studying microbial dynamics in several extreme conditions, such as alkaline or acidic pH, high temperature, water stress, and UV radiation (Figure 1). Its distinctive climate is the result of the confluence of a subtropical high-pressure zone, the cold Humboldt Current on the coast, offshore winds, as well as the Andean rain-shadow effect and latitudinal position of the region (Houston and Hartley, 2003).

### Altiplanic Ecosystems

The Andean Altiplano occupies part of Peruvian, Bolivian, and Chilean territory with a mean altitude of 3,700 m above sea level and covers an area that is 300 km wide and 1,500 km long (Muñoz and Charrier, 1996). The Altiplano is surrounded by volcanoes and mountains rising up to 6,700 m and represents one of the largest *plateaus* in the world. The Altiplano possess several extremes environments, such as thermal waters and geysers, some basins, salars, large lakes in the north and salt flats in the south. Some of the more characteristic Altiplano ecosystems are described below.

#### Parinacota Region

The paleolakes Chungará Lake, Parinacota Wetland, and Piacota Lagoon are located 4,300–4,500 m above sea level. The microbial community composition is highly variable between the different wetlands, but also between water and sediment samples (Dorador, 2007). Each of these environments supported a unique community of Bacteria and Archaea, revealing a differentiation between high altitude lakes, freshwater wetlands, and saline wetlands.

#### Geothermal Springs and Geysers

The extreme conditions (high temperature and pressure) are characteristic of hot springs and geysers located in the Northern region and is the result of the permanent interaction of groundwater with magma and hot igneous materials stemming from near the rather abundant volcanic areas (Jones and Renaut, 1997; Fernandez-Turiel et al., 2005; Rafferty, 2010).

The Geothermal site El Tatio (Kunza language, meaning “crying grandfather”) is the largest geyser field in the Southern hemisphere and one of highest geysers around the world (∼4,200 m.a.s.l.) (Glennon and Pfaff, 2003). Located on the Andean Altiplano, it harbors over 100 erupting springs and is surrounded by high volcanoes. The water discharged by the Tatio Geyser is very rich in silica, with the highest concentrations reported for a natural surface water, of both arsenic (∼0.5 mM) and antimony (∼0.02 mM) at a nearly neutral pH (Landrum et al., 2009). These properties differ from other well-studies geothermal sites, such as basins in Yellowstone National Park, United States, and Dallol Volcano, Ethiopia, for which their waters can reach extreme pH values (Rowe et al., 1973; Barbieri and Cavalazzi, 2014). Therefore, El Tatio in an environment with unique physico-chemical characteristics.

The North regions of Chile harbor many hydrothermal fields that remained less explored than El Tatio. For instance, the Surire hydrothermal system, at 4,000 m.a.s.l., is located in the south part of Surire Salar (Procesi, 2014). The water discharges are characterized by higher concentration of sulfate. Analogous situation occurs with Lirima Geothermal field that is located at an altitude of 3,900 m.a.s.l., 25 km southwest of the Sillajhuay volcanic chain. The Lirima area contains bubbling pools with temperatures between 38°C and 80°C (Tassi et al., 2010; Procesi, 2014).

### Salars

Chile harbors a huge diversity of salars along its territory. Saline ecosystems are located in Altiplano, Atacama Desert, Central Chile, and Patagonia. Specific salars that has been subjected to microbial studies will be described.

#### Athalassohaline Ecosystem: Huasco Salar

Athalassohaline systems are saline ecosystems with a non-marine origin but originated from evaporation of fresh water in a system dominated by calcium, magnesium and sulfate, in contrast with sodium and chloride that are prevalent in the ocean (Grant, 2006). At 3,800 m above sea level, Huasco Salar is a good example of an athalassohaline system (Dorador et al., 2010). These systems exhibit extreme conditions such as low temperatures and atmospheric pressure, high solar radiation, negative water balance, and a wide range of salt concentrations (Castro-Severyn et al., 2017).

#### Acidic Salars: Ignorado Salar and Gorbea Salar

Two acidic salars in Chile are located in the Andes Mountains (Risacher et al., 2003). Gorbea Salar is located in a basin with extreme acidic brines, with pH ranging from 2 to 4 and NaCl concentrations from 0.03 to 1.3 M (Quatrini et al., 2017). Ignorado Salar is an acid saline lake, with surface waters with pH ranging from 3.3 to 4.1 and a total dissolved solids concentration from 0.5 to 3% (Karmanocky and Benison, 2016).

#### Borderline Salar: Atacama Salar

Salar de Atacama is located between the Atacama Desert and the westernmost margin of the Altiplano at 2,300 m above sea level (Zúñiga et al., 1991). It is the largest and oldest evaporating basin in Chile, and the largest Quaternary halite deposit worldwide (Warren, 2010; Lara et al., 2012). Salar de Atacama has some shallow lakes with high salt concentration (Zúñiga et al., 1991) and with distinct geological features compared to the Altiplano basins (Demergasso et al., 2010).

#### Central and Southern Salars

Other saline ecosystems are also located in the Central Region and the Southern Patagonia (De Los Rios-Escalante and Gajardo, 2010; Plominsky et al., 2014). Cáhuil Lagoon is a saline ecosystem located at the coast close to Pichilemu in Central Chile (Figure 1).

### Patagonia

Patagonia is comprised by an extensive structure of cold fjords (e.g., Comau Fjord) and channels, and by oligotrophic cold lakes in Southern Chile, characterized by low temperatures, low nutrient concentration and low dissolved organic carbon (Gutiérrez et al., 2015; Aguayo et al., 2017). Cold lakes in Patagonia (e.g., Grey Lake, Figure 1) are affected by seasonal temperature variations, ranging from 4°C in winter to 20°C in summer (Aguayo et al., 2017).

Volcanic activity has been also reported with a high eruption frequency (Carrillo et al., 2018), which has formed numerous hydrothermal fields including Porcelana Hot Spring and Geyser, and Cahuelmo Hot Spring. Cahuelmo Hot Spring is a geothermal site located at the sea level coast of Cahuelmo Fjord, that contains waters rich in metallic minerals and elements such as pyrite, polonium, magnetite, and chalcopyrite (Mackenzie et al., 2013). On the other hand, Porcelana hot spring is located ∼100 m above sea level in Northern Patagonia. Porcelana is a pristine spring characterized by a rather extensive thermal gradient (∼38–69°C) and neutral pH (Alcamán et al., 2015).

### Antarctica

Antarctica displays extreme climates and environmental conditions above and below the water surface. This environment is dominated by strong gradients in temperature (-10°C to -2°C), salinity (35–150%), and irradiation (<0.1% to 1–5% UV radiation), properties highly variable and ultimately governed by air temperature and snow cover (Cirés et al., 2017). The search of new pigments, antibiotics, and enzymes has become a main research focus in the Antarctic continent (Loperena et al., 2012; Órdenes-Aenishanslins et al., 2016; Lavin et al., 2017). In spite of been the coldest continent on Earth, surprisingly, Antarctica harbors many geothermal sites.

## Thermophiles

**FIGURE 2:**
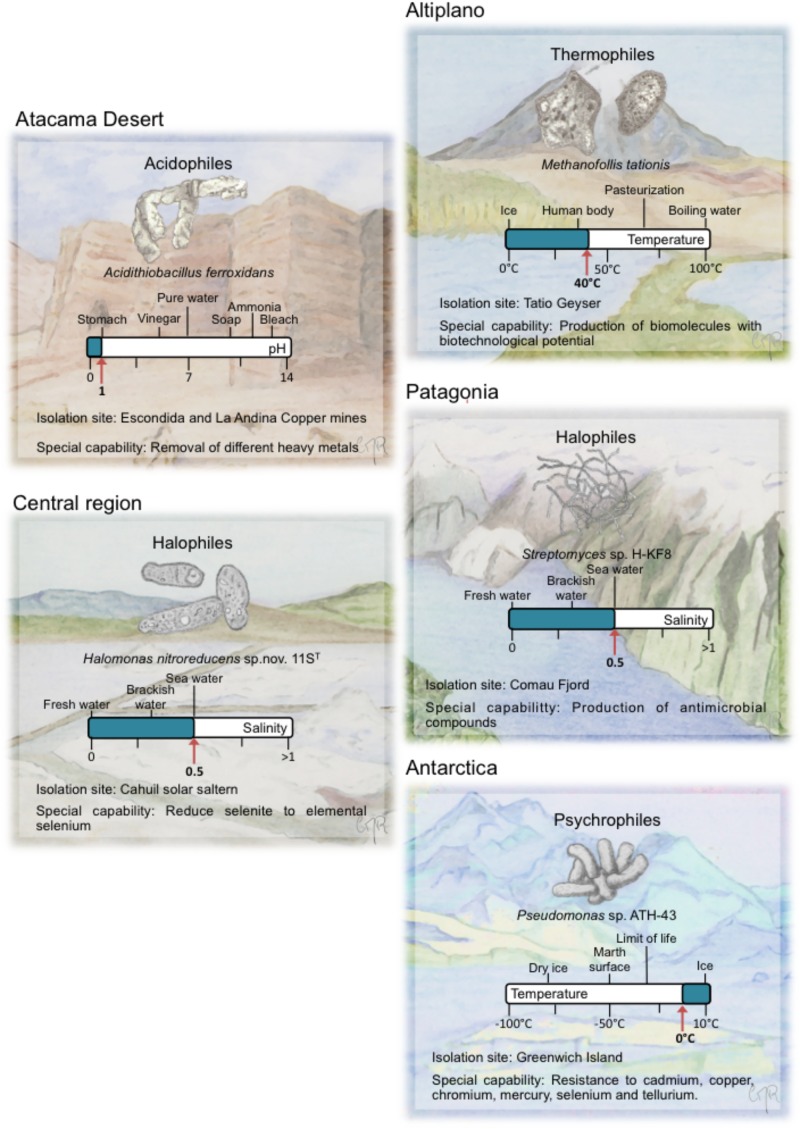
Representative extremophiles from five main biotopes in Chile. For each extremophile, a special capability is described and its ecosystem is drawn. A bar with well-known values and the isolation value for each of the extreme environmental variables is depicted.

Early life in Earth was initially dominated by thermophilic anaerobes that had chemoheterotrophic or chemolithoautotrophic metabolism capable of been sustained by hydrothermal energy sources (Woese et al., 1990; Konhauser et al., 2003; Weiss et al., 2016). Thermophiles are organisms, mainly prokaryotes, whose optimum growth temperatures are >44°C (Madigan et al., 2000). Hyperthermophiles are thermophiles that grow at temperatures >80°C (Berenguer, 2011) (Table 1). Thermophiles and hyperthermophiles are present in several natural ecosystems such as geothermal waters (Figure 2), hot springs, mud pots, fumaroles, geysers, deep-sea hydrothermal vents, volcanoes, and also in engineered environments, such as compost facilities and anaerobic reactors (Ahring, 1995; Rastogi et al., 2010; Urbieta et al., 2015b).

**FIGURE 3:**
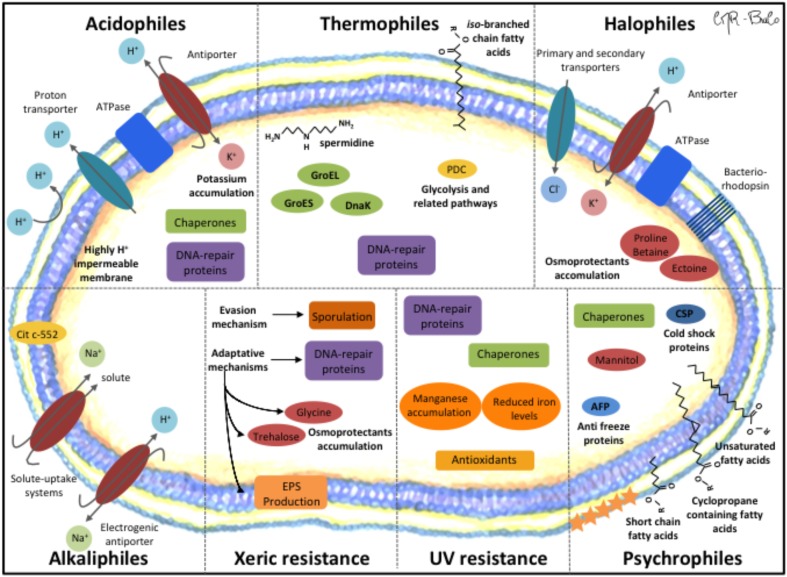
Molecular mechanisms of extremophiles for their adaptation to extreme environmental conditions. Acidophiles. (i) Potassium antiporter releases protons towards the extracellular medium, (ii) ATP synthase, (iii) membrane highly impermeable to protons, (iv) Chaperones, and (v) DNA-repair proteins. Thermophiles. (i) Upregulated glycolysis proteins (e.g., pyruvate dehydrogenase complex (PDC)), (ii) Lipids with iso-branched chain fatty acids and long chain dicarboxylic fatty acids, (iii) polyamines (spermidine), and (iv) Chaperones. Halophiles. (I) High salt-in strategy: (i) chloride transporters (primary or secondary), (ii) potassium uptake into cells by concerted action of bacteriorhodopsin and ATP synthase. (II) Low-salt strategy: (i) *de novo* synthesis or uptake of osmoprotectants (proline-betaine, ectoine) that maintain osmotic balance and establish the proper turgor pressure under different salt concentration. Psychrophiles. (i) high degree of unsaturated, cyclopropane containing fatty acids and short chain fatty acids, (ii) Cold shock proteins (CSP) (iii) Chaperones, (iv) Anti-freeze proteins (AFP) restrict the ice growth on protein surfaces, (v) Mannitol and other compatible solutes accumulate in the cell cytoplasm as cryo-protectants to prevent protein aggregation, and (vi) Carotenoids (star symbols) support maintenance of membrane fluidity and prevent cell damage by UV radiation. UV resistance. (i) Manganese accumulation and reduced iron levels, (ii) Antioxidants (glutathione), (iii) Chaperones, and (iv) DNA-repair proteins. Xeric resistance. (I) Evasion mechanism: (i) bacteria sporulation. (II) Adaptation mechanism: (i) increased extracellular polymeric substances (EPS), (ii) DNA-repair proteins, and (iii) accumulation of osmoprotectants (glycine, trehalose). Alkaliphiles. (i) Electrochemical gradient of Na^+^ and H^+^ by electrogenic antiporters for proton accumulation, (ii) Na^+^-solute uptake system, and (iii) Cytochrome c-552 enhance terminal oxidation function by electron and H^+^ accumulation.

A general mechanism of thermophiles to safeguard their cellular components at high temperature is the adaption of thermophilic proteins through amino acid changes in their primary structure, increasing their thermal stabilities (Xu et al., 2018). Thermophilic proteins possess a larger fraction of amino acid residues in α-helices and have shorter amino acid length (Urbieta et al., 2015a; Xu et al., 2018). A main mechanism in thermophiles is the role of heat shock proteins (HSPs) including the chaperones DnaK, GroEL, and GroES to assist protein folding (Figure 3). DNA-repair systems (e.g., SOS system) are also active to respond to DNA damage. For the stabilization of the membranes, thermophiles use branched chain fatty acids and polyamines (e.g., spermidine). Another mechanism of thermophiles is the use of compatible solutes to stabilize cell components (Urbieta et al., 2015a). In addition, upregulated glycolysis pathway (e.g., pyruvate dehydrogenase complex) proteins provide immediate energy to cope with heat stress (Wang et al., 2015).

An increase in the use of (hyper)thermophilic microorganisms has been observed, especially for the need of the industry to couple biological solutions at high-temperature industrial processes (Urbieta et al., 2015a). Industrial waste reactors containing living microorganisms required to be pre-cooled down due to the fact that several industrial processes are carried out at temperatures >100°C (Gavrilescu, 2010). Therefore, (hyper)thermophiles offer a suitable solution due to their capabilities to grow at such high-temperature and also resist and metabolize several pollutants from contaminated industrial wastewaters (Vieille and Zeikus, 1996). Previous studies have demonstrated the high potential of (hyper)thermophilic pure cultures and consortia for bioremediation of heavy metal-contaminated surface and groundwaters, including biosorption and immobilization of radionuclides and heavy metals (Chatterjee et al., 2010; Sar et al., 2013), removal of heavy metals (Ilyas et al., 2014) and for degradation of persistent organic compounds such as aliphatic and (poly)aromatic hydrocarbons (Mnif et al., 2014; Zhou et al., 2018), and synthetic dyes (Deive et al., 2010). Increasing research is conducted into evaluating the applications of thermostable enzymes in waste treatment and remediation (Kataoka et al., 2014; Wang et al., 2015; Rigoldi et al., 2018).

A high portion of the Chilean territory is superimposed on the subducting Nazca plate, the largest tectonic relief, that drives displacement and rearrangements of geological structures and generates routinely megathrust earthquakes (Armijo et al., 2015). In those areas, the compression and decompression of magma may modulate volcanic activity on land, in the seafloor, and in the deep subsurface, generating abundant natural formations that are suitable for survival and growth of (hyper)thermophilic microbial life forms. Geothermal fields are usually main contributors of arsenic to both surface and subsurface water (Ballantyne and Moore, 1988; Smedley and Kinniburgh, 2002). The water influx is particularly important at the water discharged at the Tatio Geyser, which achieved in surface water a arsenic concentration of 45 mg L^-1^ (Landrum et al., 2009). The effluent discharges caused high arsenic levels (0.1–1.5 mgL^-1^) throughout the basin of Loa River. Besides the potential human health risks (Yañez et al., 2005), those arsenic levels impact also the microbiota. *Pseudomonas fluorescens* and *Serratia odorifera* strains isolated from an arsenic-polluted river in the Atacama Desert showed tolerance to arsenic 800–1,000 mM (Campos et al., 2009; Escalante et al., 2009). Due to their physicochemical properties, the water discharged by the Tatio Geyser is an unique environment to study (hyper)thermophiles. A fraction of the water is very rich in silica, contains high antimony concentration (∼0.02 mM), low sulfite concentration (∼0.5 mM) at a nearly neutral pH (Landrum et al., 2009). Most of the worldwide studies efforts directed to the biotransformation of arsenic by (hyper)thermophiles have been restricted to sulfidic waters at extreme pH conditions, including high arsenic geothermal sites in Yellowstone National Park in United States and Dallol Volcano in Ethiopia (Rowe et al., 1973; Barbieri and Cavalazzi, 2014). Research has been focused toward isolation and characterization of (hyper)thermophiles tolerant to As, or capable of playing a role on its biogeochemical cycle. The geothermal water discharges arsenite As(III), which is the most mobile and toxic form of arsenic that progressively oxidizes to arsenate As(V) downstream (Engel et al., 2013). Several prospections have shown that nearly all of the geyser features and streams at El Tatio are covered by biofilms and colorful microbial mats dominated by photosynthetic bacteria (*Chloroflexi* and cyanobacterial communities) (Fernandez-Turiel et al., 2005; Engel et al., 2013; Plenge et al., 2017). Microbial community analysis based on 16S rRNA gene of the geyser-discharge water revealed that *Chloroflexi*, *Deinococcus*–*Thermus*, *Aquificales*, and *Chlorobi* were the most prevalent microorganisms in the zones where arsenite reduction takes place (Engel et al., 2013). Indeed, the survey of functional genes revealed that most of the arsenite oxidase *aio*A-like gene found within the community showed high identity to genes belonging to strains of the thermophilic anoxygenic phototroph *Chloroflexus aurantiacus*. *Chloroflexi*-like *aioA* gene sequences obtained from the microbial community of El Tatio clustered on two separated clades likely representing additional diversity that could be associated with either novel groups of arsenic-resistant bacteria, or novel mechanisms of As resistance. It is also noteworthy to mention that *Chloroflexus aurantiacus* can either grow phototrophically under anaerobiosis or chemotrophically under aerobic and dark conditions, providing enough metabolic flexibility to function as a potential strain for bioremediation of As-contaminated environments, but also during carbon sequestration efforts (Tang et al., 2011).

Besides arsenic, the microbial communities associated to El Tatio cope with a wide variety of environmental stressors, such as the presence of other toxic metals (e.g., antimony) and the impact of extreme UV radiation. Living at such selective pressure has fostered the development and retention of a suite of metabolic and physiological adaptations that has enormous potential to be used for biotechnological applications. On one hand, multi-resistant microbial isolates may be useful in bioremediation. Furthermore, a second area of interest is the identification and characterization of the mechanisms of resistance to multiple stressors. Indeed, enzymes synthesized by (hyper)thermophiles display high levels of thermostability, converting them in candidates to explore potential novel applications for industrial processes. For example, the archaeon isolated from a solfataric pool located in El Tatio, *Methanofollis tationis* (Figure 2, first described as *Methanogenium tatii*) (Zabel et al., 1984; Zellner et al., 1999) produced its own particular pterin, a type of biomolecule involved in immune modulation, cellular signaling, metabolism and pigmentation. Two years after its isolation, the identification and structure of the novel pterin produced by *Methanofollis tationis*, named tatiopterin, were elucidated. Afterward, it has been shown that tatiopterin specifically enhances photostability of materials, preventing the bleaching of photosynthetic pigments due to irradiation (Elshahawi et al., 2015). More efforts in order to understand the role of such molecules under those conditions should be further explored. Specifically, one area of interest in this field is the increase of exploration efforts of novel thermozymes that can be applied for production of biofuels from starch and lignocellulosic waste materials. Due to their complex structure, lignocellulosic materials are degraded by physicochemical treatments to obtain cellulose, hemicellulose, and lignin, which can be further treated with hydrolytic enzymes (Urbieta et al., 2015a).

Previous prospections in the Surire Salar have described the presence of arsenic-precipitating bacteria, suggesting that may be also present in the Surire hydrothermal vents (Demergasso et al., 2007). The culturable mesophiles in Lirima wetland were reported, whereas the hot spring remain unexplored (Scott et al., 2015). Special attention should be devoted in this site due to the fact that water contain high chloride and boron concentrations, becoming a source of microorganisms capable of bioremediation of boron-polluted wastewaters. Microbes associated to the hydrothermal systems such as Puchuldiza-Tuja (4,100 m.a.s.l.), Colpitas (4,000 m.a.s.l.), Apacheta (4,500 m.a.s.l.) have not been explored yet.

Porcelana hot spring in Northern Patagonia is characterized by abundant and colorful microbial mats widespread along a rather extensive thermal gradient (∼38–69°C) (Alcamán et al., 2015). The microbial community associated to Porcelana hot spring is dominated by cyanobacteria, particularly of the diazotrophic *Mastigocladus* (Stigonematales) genus. Members of the same genus were also identified as part of biofilms in Copahue geothermal field at the North west corner of Neuquén province, Argentina (Urbieta et al., 2015b), indicating its cosmopolitan character (Miller, 2007). At Porcelana hot spring, *Mastigocladus* sp. strain CHP1 was the most dominant contributor of nitrogen through nitrogen fixation (∼87% of the *nifH* gene transcripts) (Alcamán et al., 2015). Physiological studies revealed that *Mastigocladus* isolates gather a broad range of metabolic plasticity regarding the nitrogen metabolism (Alcamán et al., 2017). *Mastigocladus* sp. strain CHP1 is capable to fix nitrogen at 60°C, the highest temperature reported for the activity of N_2_-fixing filamentous cyanobacteria (Alcamán et al., 2015). Further studies should explore the metabolic versatility of *Mastigocladus* sp. strain CHP1 in the treatment of nitrate-contaminated industrial waters at high temperature.

Environments are so dynamic that surprise us. An iconographic example of such diversity is depicted by the fact that Antarctica, a continent well known as the coldest place on earth, also harbors many geothermal sites suited for the growth of (hyper)thermophile microorganisms (Flores et al., 2013). The uppermost temperatures of those sites range from 40 to 110°C, and has extremely low concentrations of nutrients, such as N and P, and high concentrations of heavy metals (Cu, Zn, Cd, Pb, and Hg) (Muñoz et al., 2011). Although environmental conditions seem to be harsh for microbial growth, recent studies have shown that the environment is suitable for thermophilic organisms. Bacteria from the *Geobacillus*, *Bacillus*, *Brevibacillus*, *Thermus* genera and uncultured sulfate reducing bacteria were abundant in a fumarole at 90–110°C from Deception Island, an active strato volcano located in the South Shetland Islands (Muñoz et al., 2011). Some of these microbes can carry metal redox interactions that have further implications for ore formation and might be capable to recover metals from ore-containing materials. For instance, the thermophilic *Geobacillus* sp. strain ID17 is capable to synthesize gold nanoparticles when exposed to Au(III). The nanoparticles were found to be intracellularly accumulated raising the potential applications in bioremediation of gold-bearing wastes (Correa-Llantén et al., 2013). The synthesis of gold nanoparticles by bacteria can be useful for diverse biotechnological and medical applications (Montero-Silva et al., 2018). *Geobacillus* species are also capable to degrade organic pollutants. Indeed, members of the same clade including *Geobacillus thermoleovorans* T80 degrade hexadecane (70%) during bioremediation at ∼60°C. *Archaea* have also been isolated from soils extracted at Deception Island. Dennett and Blamey (2016) isolated the hyperthermophilic archaea *Pyrococcus* sp. M24D13, and characterized a novel thermostable cyanide-degrading nitrilase. This thermostable enzyme may be useful to remediate cyanide contaminated waste streams (Dennett and Blamey, 2016). (Hyper)thermophiles and their novel thermostable enzymes are of considerable biotechnological interest to find novel alternatives for bioremediation and other bioprocesses to be carried out at high temperatures.

## Acidophiles

Acidophiles are defined as organisms that grow at an optimum pH < 5 (Johnson, 2008). Extreme acidophiles are microorganisms that showed an optimum growth at pH 3 or less, whereas moderate acidophiles are those that grow with optimum pH between 3 and 5. Acid-tolerant microorganisms, have optimum pH > 5, but are still active in low pH environments (Johnson, 2007, 2008). Extreme acidophilic organisms are exclusively microbial and distributed in Archaea, Bacteria, and Eukarya domains (Sharma et al., 2016).

Acidophiles maintain the cytoplasmic pH close to neutrality to safeguard the acid-labile cellular constituents, which require the generation of a large pH gradient. Three main mechanisms are involved in the adaptation to an acidic environment (Figure 3). A first mechanism is an active pumping of protons to the maintenance of ΔpH, by proton flux systems. The role of proton efflux via transport pumps in the electron transport chain alongside the influx of protons through the F_0_F_1_-type ATP synthase has been described in *Bacillus acidocaldarius*, *Thermoplasma acidophilum*, and *Leptospirillum ferriphilum* (Michels and Bakker, 1985). Additional proton flux systems include primary proton pumps (symporter) and secondary proton pumps (e.g., cation/H^+^ antiporter), and proton-consuming reactions. A carbonic anhydrase and amino acid decarboxylases that aid in pH homeostasis by consuming protons have been reported in *L. ferriphilum* (Christel et al., 2018). A second mechanism is a decreased permeability of the cell membrane to suppress the entry of protons into the cytoplasm. The influx of protons is inhibited by the inside positive membrane potential formed by K^+^ ions (Christel et al., 2018). A wide *repertoire* of genes related to cell membrane biosynthesis that may be associated with acid tolerance was identified in *L. ferriphilum*. The presence of tetrapetric lipids in the cell membrane that provide tolerance to acidic pH has been reported in *Archaea Ferroplasma acidiphilum* and *Sulfolobus solfataricus*. A third mechanism is an improved protein and DNA-repair systems in acidophiles compared to neutrophils. An external pH shift from 3.5 to 1.5 induced proteins that are involved in the heat shock response such as chaperones in the acidophile *At. ferrooxidans* (Amaro et al., 1991).

Acidophilic microorganisms play an important role in biomining of metals from low grade sulfur minerals (Bustos et al., 1993; Seeger and Jerez, 1993; Seeger et al., 1996; Okibe et al., 2003; Demergasso et al., 2005, 2010; Acosta et al., 2017). Previous studies have demonstrated the role of acidophiles in bioremediation of polluted soils and waters, through (i) metal reductive processes (Kolmert and Johnson, 2001; Suzuki et al., 2003; Leigh et al., 2015), (ii) metal adsorption onto jarosites (Natarajan, 2008; Asta et al., 2009), (iii) metal biosorption (Liu et al., 2004; Chakravarty and Banerjee, 2012), and (iv) degradation of petroleum hydrocarbons (Stapleton et al., 1998; Margesin and Schinner, 2001; Christen et al., 2012; Arulazhagan et al., 2017). Additionally, enzymes from acidophilic microorganisms are explored due to their tolerance to low pH, which favors their industrial applications in starch, fruit juices, feed and baking industries (Matzke et al., 1997; Nakayama et al., 2000; Serour and Antranikian, 2002; Sharma et al., 2012). More recently novel applications of acidophiles including electric generation have been explored (Sulonen et al., 2015; Ni et al., 2016).

Acidophilic microorganisms are present in several natural habitats, such as solfataric fields and geothermal sulfur rich sites (Sharma et al., 2016). These sites are niches for a variety of acidophilic microorganisms with unique adaptations for survival in the hostile low pH environments such as *Sulfolobus solfataricus* and *Sulfolobus acidocaldarius* (Yellowstone National Park, United States) (Brock et al., 1972). Acidophilic microorganisms have also been reported in anthropogenic environments such as acidic mine drainage (AMD), which are associated with heavy metals and coal mining. The acidophilic microorganisms mobilize metals and generate AMD. AMD generates most of the extremely acidic niches on Earth and disseminates heavy metals in the environment (Panda et al., 2016).

In Chile, an important area where acidophiles have been studied is located in the Atacama Desert. The microbial solubilization of metals in acidic environments has been successfully used in bioleaching for the extraction of metals. In Escondida mine located 170 km south-east from Antofagasta, the analyses of the microbial community of a low-grade copper sulfide leach pile indicated the presence of *At. ferrooxidans* (Figure 2), *At. thiooxidans, L. ferriphilum*, and *F. acidiphilum* (Galleguillos et al., 2008; Remonsellez et al., 2009; Demergasso et al., 2010; Acosta et al., 2017). Prokaryotic acidophile microarray (PAM) analysis showed members of *Sulfobacillus* genus in samples from heap leaching (Remonsellez et al., 2009). 16S rRNA genes phylogenetic analysis, real time PCR and metagenomics analysis revealed in the heap leaching the bacteria *At. ferrooxidans, At. thiooxidans, At. caldus, At. ferrivorans, L. ferriphilum, S. acidophilus, S. thermosulfidooxidans*, and *Acidiphilium* spp., and the archaea *Ferroplasma acidiphilum, Ferroplasma acidarmanus*, and *Sulfolobus* spp. (Demergasso et al., 2005, 2010; Soto et al., 2013; Acosta et al., 2017). *Acidithiobacillus*, *Leptospirillum*, and *Sulfobacillus* strains have also been reported in the copper tailings of La Andina mine in Central Chile (Figure 2). Molecular methods revealed the presence of heterotrophic acidophiles associated to *Acidobacterium capsulatum*, *Acidobacterium*-like bacterium and *Acidiphilum* sp. (Diaby et al., 2007). In Chile, solfataras throughout Andes Mountains (e.g., Purico Complex and Nevados de Chillán) that harbor acidophiles have been described. The moderate acidophile archaea *S. solfataricus* was isolated from a hot spring in Nevados de Chillán (Valdebenito-Rolack et al., 2017). To date, acidophilic microbes have been scarcely studied in diverse acidic environments in Chile including solfataras.

In the last decades, the bioremediation potential of acidophiles in acidic environments have been studied. Acidophiles capable to degrade phenol at low pH have been reported. The acidophile *S. solfataricus* degrades phenol (Christen et al., 2011). Zhou et al. (2016) reported the degradation by *S. acidophilus* of phenol, and methylphenols. Acidophilic bacteria able to grow in presence of alkanes have been described (Hamamura et al., 2005). Acidophiles including *Acidisphaera*, *Acidiphilium*, and *Acidithiobacillus* strains were capable to degrade 50% hexadecane in hydrocarbon-amended soil/sand mixtures. Acidophiles have been used for the treatment of petroleum-polluted wastewater under acidic condition (Arulazhagan et al., 2017). Acidophilic microorganisms such as *Acidithiobacillus* and *Sulfobacillus* strains may be useful for the remediation of hydrocarbon-polluted acidic sites (Ivanova et al., 2013).

The removal of heavy metals by acidophiles from Chile has been reported. *At. ferrooxidans* has been used for removal of different heavy metals at laboratory scale. The addition of *At. ferrooxidans* to an AMD increased precipitation kinetics of heavy metals and decreased water iron content, accelerating heavy metal removal (Darkwah et al., 2005). *At. ferrooxidans* has been used for the *ex situ* bioremediation of uranium (VI), removing up to 50% of uranium (100 mg L^-1^) from polluted mine water (Romero-González et al., 2016). Takeuchi and Sugio (2006) reported that mercury was almost completely removed by volatilization in mercury-polluted soil by *At. ferrooxidans* strains SUG 2- 2 and MON-1. Also MON-1 cells immobilized in PVA resins efficiently volatilize mercury from mercury-polluted wastewater. Bioremediation by bioaugmentation with heavy metal-resistant bacteria of mercury-polluted waters has been reported (Rojas et al., 2011). This bioremediation process was scaled up (Bravo, 2017) and may be applied in mercury-polluted sites closed to metal mining activities. *At. ferrooxidans* and *Leptospirillum ferrooxidans* have been used for arsenite removal. Bioremoval occurs through the adsorption of arsenic (III) onto the jarosites generated during microbial growth (Natarajan, 2008). Furthermore, *At. ferrooxidans* and *At. thiooxidans* strains are able to reduce chromium (VI). The almost complete removal (93%) of chromium (VI) from electroplating waste by *A. thiooxidans* has been reported (Cabrera et al., 2007). *At. thiooxidans* is able to reduce uranium by polythionates that are synthesized during oxidative sulfur metabolism (Gargarello et al., 2010). *At. ferrooxidans* has been applied for removing sulfur from solids and gases and heavy metals from electric wastes and sludge (Zhang et al., 2018). In addition, the removal of Ni and Hg from port sediments through bioaugmentation with a consortium of iron-oxidizing acidophilic bacteria (*A. thiooxidans*, *A. ferrooxidans*, and *L. ferrooxidans*) in microcosm was observed (Beolchini et al., 2009).

Metal mining is a key player in the Chilean economy. Therefore, diverse acidophiles useful for bioleaching have been isolated, characterized and applied for bioleaching in Chile. Interestingly, some of these acidophilic microorganisms have been used at laboratory scale for the bioremediation of heavy metals and organic pollutants. However, the application of these acidophiles in bioremediation at industrial scale is still an important challenge. Acidophilic microbes from extreme environments are attractive biocatalysts for bioremediation processes under acidic conditions, especially for heavy metals.

## Halophilic Microorganisms

Microorganisms belonging to the three domains of life are present over the whole range of salt concentrations in the environment (Oren, 1999). Halophiles are microorganisms that obligately require salt to grow (Margesin and Schinner, 2001). They are classified based on their optimal NaCl concentration for growth as slight halophiles (0.2 M), moderate halophiles (0.5–2.5 M), borderline extreme halophiles (>2.5–4.0 M) or extreme halophiles (>4.0–5.9 M) (Edbeib et al., 2016). Halophilic microorganisms are ubiquitous in salars, saline lakes, oceans, polar ice, and coastal areas (Edbeib et al., 2016). Halotolerants are microorganisms that grow in the presence and absence of NaCl, and those that grow in presence of >2.5 M NaCl are considered extremely halotolerant (Margesin and Schinner, 2001).

Halophilic and halotolerant microorganisms have adapted and evolved to survive in saline environments. They gather unique metabolic properties toward maintaining more water in the cytoplasm than in their surroundings, avoiding water losses. Halophilic or halotolerant microorganisms have evolved two main strategies (Figure 3). The first strategy is maintaining an intracellular salt concentration equivalent to the environment, and consequently, all intracellular systems have been adapted. This is achieved with chloride and potassium uptake into the cells by transporters (primary or secondary) and concerted action of bacteriorhodopsin and ATP synthase. The other strategy is maintaining low intracellular salt concentration, and therefore osmotic pressure is balanced by organic compatible solutes, such as betaine and ectoine. Due to its nature, the latter strategy does not require a global adaptation of the intracellular machinery (Oren, 1999; Margesin and Schinner, 2001).

Chile gathers several high saline environments. Most of them are concentrated in the Northern region, where 52 saline lakes and salt crusts are distributed alongside The Andes, spanning an area of over 200,000 km^2^ of the Atacama Desert. The saline ecosystems found in the Northern region can be classified in (i) Borderline Salars, (ii) Athalassohaline ecosystems, which are located in the Altiplano, and (iii) Acidic Salars (Risacher et al., 2003; Risacher and Fritz, 2009). Particularly, in Lejía Lake, an extreme saline lake nested at the base of Lascar Volcano in the Chilean Altiplano, two halotolerant bacteria closely related to *Halomonas alkaliantarctica* strain CRSS and capable to grow at 15% NaCl were isolated (Mandakovic et al., 2018). Other saline lakes are also located in Central region (Cáhuil Lagoon), and the Southern Patagonia (De Los Rios-Escalante and Gajardo, 2010; Plominsky et al., 2014). One third of bacterial isolates from different niches in the Atacama Salar were classified as moderate halophilic and halotolerant bacteria. These isolates belong to *Marinomonas*, *Vibrio*, *Alteromonas*, *Marinococcus*, *Acinetobacter*, *Micrococcus*, *Bacillus*, *Pseudomonas, Deleya*, *Staphylococcus* genera and also some Cyanobacteria, such as members of the *Cyanothece*, *Gloeocapsa*, and *Gloeobacter* genera (Zúñiga et al., 1991; Dorador, 2007). Saltern ecosystems are commonly dominated by Archaea, where members of the family *Halobacteriaceae* are the most common halophiles (Oren, 2002). Metagenomic studies performed on samples extracted from a salt crystallizer pond located in the Cáhuil Lagoon, revealed that 61% of the gene sequences belonged to Archaea, mainly from the *Halobacteriaceae* family, followed by 19% sequences from viruses and 16% from bacteria (Plominsky et al., 2014). Microbial communities in the Atacama Desert ecosystems, such as halites, saline soils and salt lakes, are also dominated by members of the *Halobacteriaceae* family (Robinson et al., 2015; Finstad et al., 2017).

Arsenic is a metalloid that is widely distributed at high concentrations along the Atacama Desert. The sources of arsenic are often associated with volcanic-hydrothermal springs, metal ores and anthropogenic activities. Microbial communities from Atacama Salar contributed to arsenic reduction (Lara et al., 2012), which is a process that has been widely studied in saline-alkaline systems (Oremland et al., 2004; Kulp et al., 2007). *Shewanella* sp. strains Asc-3 and CC-1 isolated from Ascotán low-pH salt flats (Demergasso et al., 2007; Lara et al., 2012) were capable of precipitating arsenic (Table 2). Surprisingly, arsenic-precipitating bacteria accounted for 50% of the bacterial communities, suggesting that arsenic-based processes in this ecosystem are significant in the formation of arsenic minerals (Demergasso et al., 2007).

**Table 2:** Halophiles isolated from diverse extreme environments in Chile and their bioremediation potential.

Strain	GenBank accession number	Isolation source	Salt tolerance (M)	Bioremediation potential	Reference
*Haloferax* sp. CL47	HQ438281	Cahuil Lagoon	3.4	Naphthalene, anthracene, phenanthrene, pyrene, and benzanthracene degradation	Bonfá et al., 2011
*Halomonas nitroreducens* 11S^T^	EF613113	Cahuil Lagoon	0.5–3.4	Selenite reduction	González-Domenech et al., 2008
*Shewanella* sp. Asc-3	EF157293	Ascotán salt flat	0–0.5	Arsenic precipitation	Demergasso et al., 2007
*Shewanella* sp. CC-1	EF157294				
*Exiguobacterium* sp. SH31	LYTG01000000	Huasco Salar	0.4	Arsenic resistance	Castro-Severyn et al., 2017
*Microbacterium* sp. CGR1	CP012299	Alto Andino, Atacama Desert	0–1.2	Arsenic resistance	Mandakovic et al., 2015


*Exiguobacterium* sp. SH31 isolated from a moderate saline environment in Huasco Salar was able of grow in presence of As(III) 10 mM and As(V) 100 mM, highlighting its natural resistance to arsenic. *Exiguobacterium* strains capable of reducing arsenate and chromium have been reported. Interestingly, *Exiguobacterium* strains have been explored for bioremediation applications. An *E. aurantiacum* strain isolated from a lake in Southern Spain degrades pesticides (López et al., 2005). *Microbacterium* sp. CGR1 (Table 2) from the Atacama Desert tolerates NaCl 1.2 M, and possesses arsenic resistance (Mandakovic et al., 2015). An additional survey of metal resistance in halotolerant and halophilic isolates from the Atacama Desert showed that most of them were capable to cope with the presence of heavy metals such as Cd, Zn, Ni, Cu, and Co. For example, *Thalassobacillus devorans* showed high tolerance to cadmium and nickel (Moreno et al., 2012).

Other Chilean ecosystems such as Cahuil marine salterns are sources for strains capable to be used in bioremediation platforms. The extreme halophile *Haloferax* sp. CL47 that was isolated in Cahuil degrades several polyaromatic hydrocarbons (PAHs) such as naphthalene, anthracene, phenanthrene, pyrene and benzanthracene (Bonfá et al., 2011). In addition, *Haloferax* sp. CL47 has been used for petroleum degradation in wastewater. The extreme halophile *Halomonas nitroreducens* 11S^T^ (Figure 2 and Table 2) showed the capability to reduce selenite to elemental selenium (González-Domenech et al., 2008).

Halotolerant strains belonging to the phylum *Actinobacteria* were isolated from marine sediment samples from the Comau Fjord in Northern Patagonia (Undabarrena et al., 2016). These strains showed tolerance to up to NaCl 1.7 M. *Streptomyces* sp. H-KF8 (Figure 2) tolerates different heavy metals, such as Ni (15 mM), Cu (0.75 mM), Co (6 mM), Zn (50 mM), Cd (1.5 mM), Hg (60 μM), Te (40 μM), Cr (20 mM), and As (100 mM). The genome sequencing of the *Streptomyces* sp. H-KF8 strain revealed the presence of 49 heavy metal resistance genes (Undabarrena et al., 2017).

Due to industrial activities or natural sources, saline environments frequently have a high concentration of organic compounds (Castillo-Carvajal et al., 2014; Edbeib et al., 2016). Industrial wastewaters frequently possess high salt concentration, and high levels of organic matter and pollutants, for which a wide variety of conventional biological treatments are not currently suitable (Bonfá et al., 2011). The presence of high levels of aromatic compounds in saline wastewater during crude oil extraction has been described (Le Borgne et al., 2008). The biodegradation of hydrocarbon-derived pollutants in both soils and groundwater is impaired under elevated salt concentration (Lay et al., 2010). Thus, the application of halotolerant pollutant-degrading bacteria is essential for the bioremediation processes in saline environments.

Halophilic microorganisms capable of degrading hydrocarbons, lignocellulosic materials, chlorophenols, formaldehyde, nitroaromatic compounds have been reported (Oren et al., 1992; García et al., 2004). The halophile *Halomonas organivorans* degrades a wide range of aromatic compounds (García et al., 2004). *Halomonas* sp. KHS3 is capable to degrade diverse aromatic hydrocarbons and to produce extracellular rhamnolipids (Corti Monzón et al., 2018). Rhamnolipids are biosurfactants that are used for bioremediation and enhanced oil recovery (Rahman et al., 2003; Sachdev and Cameotra, 2013; Sekhon Randhawa and Rahman, 2014). Additionally, halophilic microorganisms capable of degrading lignocellulosic and nitroaromatic substrates, and chlorophenols have been reported (Oren et al., 1992). The applications of halotolerant bacteria for bioremediation of natural and industrial saline environments is an attractive challenge (Edbeib et al., 2016).

Climate change, chemical fertilizers and saline water used for irrigation are increasing salinity in agricultural soils (Valipour, 2014). Salt toxicity is a major restrictive factor in crop productivity and 20% of the cultivated land worldwide are seriously affected by salinity (Zhu, 2001; Farhangi-Abriz and Nikpour-Rashidabad, 2017). In response to salt stress, reactive oxygen species (ROS) are accumulated in plant tissues, damaging the photosynthetic apparatus and cellular membranes (Bose et al., 2014; Oukarroum et al., 2015). For preserving osmotic and ionic homeostasis under salt stress, plants accumulate osmolytes such a glycine betaine and proline (Farhangi-Abriz and Torabian, 2017). Halophilic and halotolerant microorganisms produce glycine betaine (Lamark et al., 1991) that may protect plants in saline soils. More recent studies have shown that other halotolerant bacterial mechanisms are also involved in plant protection: (i) increase production of extracellular hydrolytic enzymes (Rohban et al., 2009), (ii) increase activity of 1-aminocyclopropane-1-carboxylic acid (ACC) deaminase that reduced plant ethylene levels, which are typically increased by salt stress (Siddikee et al., 2010), (iii) increase indole-3-acetic acid (IAA) levels that enables plant to increase nutrient uptake under salt stress (Vacheron et al., 2013). These studies support the potential application of halotolerant plant growth promoting bacteria to protect crops in saline soils.

## Alkaliphiles

Alkaliphiles are organisms that grow on alkaline habitats (pH > 9), usually showing an optimal growth within pH ∼10 (Horikoshi, 1999). These extremophiles are classified in two main physiological groups: obligate and facultative alkaliphiles. Facultative alkaliphiles are capable of growing in the pH range of 7.0–9.5, whereas obligate alkaliphiles (e.g., *Bacillus krulwichiae*) showed an optimal growth between pH 10.0 and 12.0 (Krulwich and Guffanti, 1989). Alkaliphiles may coexist with neutrophilic microorganisms under mild basic pH conditions, and also live in specific extreme environments.

To cope with high pH, alkaliphile bacteria possess molecular mechanisms, which comprise the activation of both symporter and antiporter systems (Figure 3). Electrochemical gradient of Na^+^ and H^+^ is produced by electrogenic antiporters, and the symporter system allows the uptake of Na^+^ and other solutes into the cells (Krulwich, 1995; Krulwich et al., 1998). The function of cytochrome *c*-552 in electron and H^+^ accumulation enhances the function of terminal oxidation in respiratory system (Matsuno and Yumoto, 2015). These systems enable the influx of protons and solutes inside the cell due to the alteration in the distribution of ions (e.g., Na^+^), maintaining the hydrosaline homeostasis and thermodynamic stability of the cell. The transporters are controlled, probably by signaling from a transmembrane pH sensor (Krulwich, 1995).

Diverse alkaliphilic microorganisms, including bacteria belonging to *Bacillus, Micrococcus*, *Pseudomonas*, and *Streptomyces* genera and eukaryotes, such as yeast and filamentous fungi, have been isolated from alkaline environments, including highly alkaline hyper-saline lakes (e.g., Lake Natron, Tanzania) and alkaline soda lakes (e.g., Lake Mono, CA, United States) (Duckworth et al., 1996; Groth et al., 1997; Horikoshi, 1999; Yakimov et al., 2001). Alkaliphiles are also present in highly alkaline enrichments generated by industrial activities (e.g., indigo dye plants) or soils with high alkalinity (e.g., estuaries with long periods of evaporation, clay particles with highly abundant alkaline crevices) (Grant, 2003; Sorokin et al., 2006).

Alkaliphilic microorganisms have been studied as novel sources for several biotechnological applications, including the treatment of highly toxic wastewater (e.g., dye-containing effluents). Textile effluents are characterized by the presence of high salt and alkaline pH along with the presence of toxic dyes (Maier et al., 2004; Khalid et al., 2012; Prasad and Rao, 2013). Several alkaliphiles have been isolated from these hostile environments and explored as biocatalysts for the treatment of dye-containing effluents (Hou et al., 2017). *Nesterenkonia lacusekhoensis* EMLA3 that was isolated from a highly alkaline textile effluent (pH ∼13) degrades the toxic azo dye methyl red in the presence of high salt concentration and heavy metals (Ni (II), Cr (VI), and Hg (II) (Bhattacharya et al., 2017). Alkaliphilic microorganisms have been applied to remove ammonia from N-rich saline wastewater, mainly produced by coke plants, fuel refining and fertilizer industries. Chemolithoautotrophic alkalophilic microorganisms have been applied to clean up ammonia pollution in effluents (Sheela et al., 2014; Cui et al., 2016). Complete ammonia oxidation into nitrate by bacteria could be useful for waste water treatment and to engineer ecosystems (Lawson and Lücker, 2018).

In Chile, alkaliphiles have been isolated mainly in the Northern Region, specifically along salt deposits from Atacama Desert and Altiplano. This region has aridic to semi-aridic climate regimes and comprises many different (hyper)saline deposits, including evaporitic basins, known as saltflats, and athalassohaline ponds (Chong, 1984; Demergasso et al., 2004). Microorganisms in saltflats from Llamará Salar (Central Depression), Atacama Salar (Pre-Andean Depression), Ascotán Salar and Huasco Salar (Altiplano) have been reported (Demergasso et al., 2004; Dorador et al., 2008; Castro-Severyn et al., 2017), revealing insights of the special metabolic capabilities of these alkaliphiles and their responses to multiple stressors. Mandakovic et al. (2018) isolated from Lejía Lake in the Chilean Altiplano *Microbacterium sp*. CGR2, *Planococcus sp*. strains ALS7 and ALS8 that are tolerant to pH 12. However, the extremophiles from several alkaline ecosystems in Chile have not been explored. For example, Amarga Lake (Torres del Paine National Park, Patagonia) is a shallow cold lake with hypersaline water and an alkaline pH of 8.9 (Solari et al., 2004), which should harbor polyextremophile bacteria. However, to date in Amarga Lake only the presence of stromatolites has been reported.

Several heavy metal-resistant alkaliphiles have been isolated in Chile. The microbiota associated to the athalassohaline ecosystem Huasco Salar in the Atacama Desert showed resistance/tolerance to copper, tellurium, and arsenic (Castro-Severyn et al., 2017). *Exiguobacterium* isolates from this site carry a number of stress-related genetic determinants, including metal/metalloid resistance genes. For example, the arsenic resistant strain *Exiguobacterium* sp. SH31 has a set of genes encoding proteins required for arsenic resistance, including the arsenic efflux pump Acr3, growing in presence of arsenite (10 mM) and arsenate (100 mM) (Ordoñez et al., 2015; Castro-Severyn et al., 2017). Dorador et al. (2008) analyze ammonia-oxidizing bacteria (AOB) from four sites in Huasco Salar. A phylotype exhibited 98% sequence similarity to the extremely alkalitolerant ammonia-oxidizing *Nitrosomonas europaea/Nitrosococcus mobilis* (Squeo et al., 2006; Dorador et al., 2008). AOB play a key role in the nitrogen cycle. *Nitrosomonas* and *Nitrosococcus* genera belong to Nitrosobacteria that are AOB involved in the aerobic oxidation of ammonia into nitrite in agricultural soils (Hernández et al., 2011).

Alkaliphiles have been also found in unusual habitats. Quiroz et al. (2015) reported the presence of *Halomonas alkaliphila* in a brine shrimp *Artemia* that was collected from salty lagoons scattered in saltflats of the Atacama Desert (Browne, 1980; Abatzopoulos et al., 2002; Riddle et al., 2013; Quiroz et al., 2015). *Halomonas alkaliphila* is an alkaliphilic halotolerant bacterium that grows aerobically at pH 9, which has been previously isolated from a salt pool located in Montefredane, Italy (Romano et al., 2006). Recently, two *Halomonas* strains were isolated from Lejía Lake soil in Atacama Desert (Mandakovic et al., 2018). Due to its adaptation to a wide range of salt concentrations and alkaline pH, *Halomonas* may be useful for the clean up of polluted saline habitats (Berendes et al., 1996). Additional studies of novel alkaliphilic bacteria and archaea are required to understand their metabolism and physiology and to assess their bioremediation potential.

## Microbes in Dry Environments

An increase of dry environments in diverse regions due to low rainfall, high temperatures and drought has been associated to Climate change (Mukherjee et al., 2018). Water is essential for all living organisms (Robinson et al., 2015; Lebre et al., 2017). Arid environments like deserts are considered to be at the dry limit for life (Navarro-González et al., 2003; Bull and Asenjo, 2013). Extremophile xerotolerant organisms can survive in dry environments with water activity < 0.75 (Connon et al., 2007; Lebre et al., 2017). Water activity is calculated as the ratio of the vapor pressure in an environment relative to pure water, and it is considered to be the amount of water available to organisms in the environment (Lebre et al., 2017). Arid areas are biomes with a ratio of mean annual rainfall to mean annual evaporation of <0.05 mm year^-1^, and <0.002 mm year^-1^ for extreme hyper-arid areas (Mohammadipanah and Wink, 2016). In addition to the low rainfall, other factors such as high and low temperatures, low water activity, high salinity, low concentrations of organic carbon and intense radiation intensify the xeric conditions, constraining the survival of microorganisms (Dose et al., 2001; Crits-Christoph et al., 2013).

Xerotolerant bacteria have developed two strategies to survive in dry environments: evasion of environmental stress and adaptative mechanisms (Figure 3). Evasion of dry environmental implies the conversion of cells into a state of non-replicative viability through the formation of spores (Crits-Christoph et al., 2013). Adaptative mechanisms are associated to preventing water loss and increasing water retention through the accumulation of osmoprotectants (trehalose, *L*-glutamate, glycine betaine), production of extracellular polymeric substances (EPS), modifications on the cell membrane to retain intracellular water, and synthesis of DNA-repair proteins (Dose et al., 2001; Lebre et al., 2017).

The Atacama Desert is the oldest desert on Earth with hyper-arid soils with mean rainfall <5 mm year^-1^ to 2.4 mm year^-1^ in the Yungay sector (Warren-Rhodes et al., 2006; Azua-Bustos et al., 2015). Nevertheless, microorganisms are capable to inhabit this extreme environment. Previous studies using culture-dependent methods have reported low numbers of bacteria in Atacama Desert soils, ranging from not detectable to 10^6^ CFU per g of soil, with a high degree of spatial heterogeneity (Crits-Christoph et al., 2013). Analysis through culture-independent methods on the subsurface layers of the hyper-arid core showed limited abundance of microbial communities including *Proteobacteria, Actinobacteria, Cyanobacteria, Chloroflexi, Firmicutes, Gemmatimonadetes*, *Planctomycetes*, and *Thermomicrobia* phyla (Dong et al., 2007; Crits-Christoph et al., 2013; Azua-Bustos et al., 2015; Piubeli et al., 2015; Azua-Bustos et al., 2017).

*Proteobacteria* from Atacama Desert showed a high bioremediation potential. *Pseudomonas arsenicoxydans* strain VC-1 isolated from arsenic-polluted site is able to tolerate high concentration of As(III) (5 mM), and also capable of oxidizing As(III) to As(V) at high rates (Campos et al., 2010). *Actinobacteria* are the most abundant culturable phylum present in Atacama Desert soils, including strains from *Streptomyces, Nocardia, Microlunatus, Prauserella, Microcella, Arthrobacter, Cryobacterium, Frigobacterium, Dietzia, Nocardioides, Propionibacterium, Luteococcus, Kocuria*, and *Patulibacterium* genera (Bull and Asenjo, 2013). Actinobacteria plays a relevant ecological role including resistance to heavy metals, recycling of substances, degradation of complex polymers and organic persistent pollutants, and synthesis of bioactive molecules (Claverías et al., 2015; Alvarez et al., 2017; Undabarrena et al., 2017), which may explain its capability to still predominate in such harsh environmental conditions. The extreme desiccating condition of deserts has been the main driving force in the evolution of several desert-derived Actinobacteria, which showed a wide range of biotechnological applications including the removal of organic and inorganic pollutants (Mohammadipanah and Wink, 2016; Alvarez et al., 2017). Chilean desert-Actinobacteria research has focused on obtaining microorganisms able to synthesize novel bioactive molecules (Bull and Asenjo, 2013; Azua-Bustos and González-Silva, 2014). Xerotolerant *Streptomyces bulli* and *S. atacamensis* synthesize ansamycin and 22-membered macrolactones with antibacterial and antitumor activities (Santhanam et al., 2013; Mohammadipanah and Wink, 2016). However, the potential of Atacama Desert-Actinobacteria toward bioremediation of pollutants and waste treatment remained underexplored.

As Atacama Desert conditions are too dry to support higher plants and most other photoautotrophic microorganisms, it gathers multiple features to ensure proliferation of primary producers. It has been shown that translucent rocks served as “refuge” for photosynthetic microbial communities, that are capable to colonize the underside of the rocks, where sufficient moisture depending of fog and dew is retained, and light intensities are not lethal. Indeed, hypolithic cyanobacteria are present in small spatially isolated islands on hyper arid Atacama Desert soil (Dong et al., 2007). These hypolithic *Cyanobacteria* communities are composed mainly of the *Chroococcidiopsis* genus, which is one of the most primitive representant of the phylum (Warren-Rhodes et al., 2006). *Chroococcidiopsis* strains are members of the hydrocarbon-degrading biofilms during bioremediation of hydrocarbon pollutants in aquatic environments (Al-Bader et al., 2013). Although Atacama *Cyanobacteria* has not been used in bioremediation processes, the use of xerotolerant bacteria of dry environments is an attractive strategy for bioremediation under hydric resource limitation.

Desertification and the demand for residential space in sites with water availability limitation have caused an increase of pollution in dry environments. Therefore, technologies for the bioremediation of polluted dry sites are required. Bioremediation of oils spills in desert soils has been reported (Godoy-Faúndez et al., 2008; Benyahia and Embaby, 2016). Strategies to alleviate the xeric stress are relevant to improve bioremediation processes in dry environments. Hereby, xerotolerant bacteria play a crucial role for bioremediation of soils in extreme dry conditions.

## UV- and Gamma-Resistant Microorganisms

Ultraviolet (UV) light is radiation with a wavelength between 40 and 400 nm. UV radiation is divided into five different ranges: (i) Vacuum UV (40–190 nm), (ii) Far UV (190–220 nm), (iii) Short-wavelength UVC (220–290 nm), (iv) Medium-wavelength UVB (290–320 nm), and (v) Relatively long-wavelength UVA (320–400 nm) (Marizcurrena et al., 2017). Life without an assured level of radiation is impossible (Pikuta et al., 2007). However, this type of radiation can be one of the most detrimental abiotic factors causing serious damages in organisms at community and cellular levels. On one hand, UV light constrains the microbial diversity and dynamics of ecosystems to only those UV-resistant (micro)organisms. On the other hand, UV radiation can affect cell survival, producing DNA damage and mutations, oxidative stress and protein denaturation (Marizcurrena et al., 2017; Pérez et al., 2017). UVB and UVC are the most harmful radiation to life (Paulino-Lima et al., 2016). UVB causes direct and indirect DNA damage. UVC may cause direct DNA damage by the formation of pyrimidine dimers and pyrimidine photoproducts (Marizcurrena et al., 2017). However, UVC is completely filtered by the atmosphere and does not reach the Earth’s surface. UVB is mostly filtered by the atmosphere, but in places where the ozone layer is thinner, the protective filter activity of the atmosphere is progressively reduced, causing a higher penetration of UVB radiation (Yang et al., 2008).

In Chile, several places have exposure to extreme solar radiation levels and high doses of UVB due to the ozone layer hole (Cordero et al., 2016; Paulino-Lima et al., 2016; Pérez et al., 2017). Atacama Desert is characterized by its high altitude, prevalent cloudless conditions, extreme dryness, relatively low columns of ozone and water vapor and intense solar UV radiation (Cordero et al., 2016; Paulino-Lima et al., 2016), which converts this desert in a key region for international astrobiology studies. The microorganisms that are capable of living under these extreme conditions are known as UV resistant extremophiles (Gabani et al., 2014). The study of microorganisms living in these hostile environments is currently focused to gain understanding on the origin of life and early evolution on Earth, to develop models for predicting consequences of future Climate change and to exploit their potential for biotechnological applications (Pérez et al., 2017).

Ultraviolet resistant extremophiles have developed different strategies to resistant UV stress (Figure 3). These strategies are related to efficient machinery for DNA-repair, induction of chaperones and active defense against UV-induced oxidative stress (e.g., glutathione accumulation) (Pérez et al., 2017). The capability of these microorganisms to repair DNA damage has been associated to radiation resistance, since it has been suggested that radiotolerant bacteria accumulate high intracellular manganese and reduced iron levels (Pikuta et al., 2007), conferring them resistance to UV radiation (Paulino-Lima et al., 2016). These types of microorganisms are usually polyextremophiles, since it has been noticed that DNA damage that accumulates during desiccation is critical also for desiccation tolerance (Mattimore and Battista, 1996).

Novel species of the genus *Deinococcus* has been isolated, mainly from environments with high UV radiation, such as deserts and arctic zones (Hirsch et al., 2004; Rainey et al., 2005; Bruch et al., 2015; Guerra et al., 2015). Strains isolated from these environments have been proposed as biocatalysts for bioremediation of radioactive waste sites (Daly, 2000; Mrazek, 2002; Ghosal et al., 2005). UV resistant strains of the *Deinococcus* genus have been associated to radiation resistance (Cox and Battista, 2005). *D. radiodurans* has been engineered for bioremediation of heavy metals and persistent organic pollutants (Daly, 2000; Cox and Battista, 2005). *Deinococcus peraridilitoris* strains KR-196, KR-198 and KR-200t (Table 1) were isolated from an arid desert soil collected in the north of Antofagasta, Chile. These isolates showed a high tolerance to ionizing radiation > 10 kGy, radiation even higher than reported for the model *Deinococcus radiodurans* strain R1 (10 kGy) (Cox and Battista, 2005; Rainey et al., 2007). *Deinococcus* sp. UDEC-P1 was isolated in Témpanos Lake, an oligotrophic lake in the Chilean Patagonia (Guerra et al., 2015). Due to their natural resistance to high levels of radiation, *Deinococcus* species isolated in Chile are potential candidates for bioremediation of radioactive wastes and environments.

High altitude lakes have the potential to be fertile niche for the isolation of novel UV-resistant microorganisms with potential for bioremediation processes. Escudero et al. (2007) have studied the diversity of microorganisms of high lakes Aguas Calientes (5,870 m above sea level) and Licancabur (5,916 m above sea level) located in the Northern Andes of Chile. These areas have high rates of UVB radiation (48.5 MW/cm^2^). Analysis of sediments and brine of Aguas Calientes lake show that the microorganisms belong mainly to the phylum *Proteobacteria*, and 70% of the sequences could not be assigned to a phylogenetic group. Licancabur Lake showed a dominance of *Cytophaga–Flavobacteria–Bacteroidetes* and *Proteobacteria*. Plankton analysis of Licancabur Lake shows poor diversity and abundance in water samples, reporting *Cyanobacteria*, *Chrysophyceae*, *Euglenophyta*, and *Chlorophyceae* strains (Albarracín et al., 2015). In spite of these pioneering reports, the microbial communities and UV-resistant microorganisms of Chilean sites with extreme solar radiation levels such as high altitude deserts and lakes and Antarctica are still mainly unknown.

## Psychrophiles

Psychrophiles refer to microbes that are capable to grow at temperatures ranging from -20 to 20°C, with an optimal growth temperature of <15°C (Morita, 1975; Clarke et al., 2013). Psychrophilic bacteria possess diverse molecular mechanisms to survive at low temperatures (Figure 3). On one side, an increase of unsaturated fatty acids, cyclopropane-containing fatty acids and short chain fatty acids in membranes prevents the loss of membrane fluidity (Feller and Gerday, 2003; D’Amico et al., 2006). A second mechanisms is the high synthesis of cold-shock proteins (CSPs) and chaperones that protect the synthesis of RNA and proteins (Godin-Roulling et al., 2015). A third mechanism is the synthesis of anti-freeze proteins (AFPs) that binds to ice crystals and generates a state of thermal hysteresis (Kristiansen and Zachariassen, 2005; Muñoz et al., 2017). A fourth mechanism is the accumulation of mannitol and other compatible solutes as cryo-protectants to prevent cell damage by UV radiation. An additional mechanism is the transport of compatible solutes such as mannitol to stabilize the cytoplasmatic environment and prevent ice formation (Baraúna et al., 2017). Nevertheless, the most general mechanism is the adaptation of psychrophilic enzymes through inherent modifications of their primary structure. Some of these amino acid changes are minimal in comparison with mesophilic proteins, playing a role in catalytic regions or stabilization of the proteins (Feller and Gerday, 2003; D’Amico et al., 2006; Cavicchioli et al., 2011).

In Chile, psychrophiles have been isolated from several cold environments, such as the high mountains of Los Andes Mountain, vast ice regions, such as the Northern and Southern Ice Field, Southern extreme zones, such as the Patagonia, and the Antarctic continent. Antarctica became a subject of intense research in several fields, including the exploration of novel microbes and their pigments, antibiotics and enzymes. The development of special research programs that supports long-timed expedition logistics have facilitated the access of researchers and increase the research on the Antarctica (Loperena et al., 2012; Órdenes-Aenishanslins et al., 2016; Lavin et al., 2017). However, in-depth research conducted on psychrophiles in cold environments, such as a report in Alpine glacier (Ferrario et al., 2017), remain to be conducted.

Antarctica plays also a huge role in two mainstream research topics, global Climate change and global contamination assessment. The pollution in Antarctica is partly explained by the grasshopper effect: pollutants are moved across the globe in the atmosphere without suffering major changes and are later deposited in continuous-forming ice sheets across Antarctica surface. Other pollution source on Antarctica resides on human activity from military and scientific bases (ASOC, 2007). The detection of diverse organic and inorganic contaminants in Antarctic territory is of increasing concern. Since Antarctica is a region where the introduction of foreign microorganisms is forbidden, the only strategy for bioremediation processes is the application of native microbes (Poland et al., 2003; Gran-Scheuch et al., 2017).

Bacteria capable to degrade crude oil at low temperatures even below the freezing point have been reported (Gerdes et al., 2005; Bowman and Deming, 2014). Alkane hydroxylases are key enzymes for the degradation of alkanes, activating them through conversion into alcohols (van Beilen and Funhoff, 2007; Fuentes et al., 2014). A survey of alkane hydroxylases genes in psychrophilic bacteria reveals the adaptability of these proteins, giving valuable insights on the degradation of long chain hydrocarbons at low temperature. Mostly enzymes were related to the AlkB and cytochrome P450 alkane hydroxylases, but also LadA and AlmA enzymes involved in long-chain alkanes degradation were reported (Bowman and Deming, 2014). The primary structure of psychrophile enzymes differ from their mesophile homologs in the preferences for specific amino acids and increased flexibility on loops, bends, and α-helices. Notably, various novel genes were reported from *Psychrobacter, Octadecabacter, Glaciecola, Terriglobus and Photobacter* genera (Bowman and Deming, 2014). Obligated hydrocarbonoclastic microorganisms (i.e., microbes that use exclusively hydrocarbons as carbon sources) isolated near to Antarctic coast have been described. For example, *Oleispira antarctica*, whose genome reveals a diversity of alkane degradation genes and a system of proteins that acts as cold barrier to circumvent low temperature effects that could stop hydroxylase reaction (Kube et al., 2013).

Studies performed in the Chilean Antarctic territory are mainly focused on Fildes Peninsula, where the human activity in scientific and military bases had caused several contamination events, including hydrocarbon spills. The estimation of the polluted area impacted is about 3.5 km^2^, corresponding to 12% of the total area of the Peninsula (ASOC, 2007). However, bioremediation plans for these sites have not yet been reported. Remarkably, the hydrocarbon degradation potential of native microorganisms from the near Argentinian Carlini Station has been described. *Polaromonas naphthalenivorans*, a strain capable of naphthalene degradation and N_2_ fixation, and strains belonging to *Nocardioides* genus were dominant in soils with higher hydrocarbon pollution (Vázquez et al., 2017). The hydrocarbonoclastic strains *Rhodococcus* sp. D32AFA, *Pseudomonas* sp. E43FB and *Sphingobium* sp. D43FB that degrade phenanthrene were isolated from diesel contaminated Antarctic soils (Gran-Scheuch et al., 2017). The Antarctic soil strain *Streptomyces* sp. So3.2 is able to produce a biosurfactant, which may be useful for hydrocarbon bioremediation (Lamilla et al., 2018).

Psychrophiles seem to be naturally adapted to cope with a wide-range of heavy metals in the environment (De Souza et al., 2006; Tomova et al., 2015; Gonzalez-Aravena et al., 2016). This adaptive trait may be explained by the presence of heavy metals in Antarctica’s sediments. In addition a high occurrence of plasmids in Antarctic bacteria has been reported, which are probably related to the transfer of resistance/tolerance genes to heavy metals such as mercury, tellurium, cadmium, copper, chromium, and lead (Mangano et al., 2013; Gonzalez-Aravena et al., 2016; Rodriguez-Rojas et al., 2016a). Interestingly, a high proportion of these bacterial isolates are multiple heavy metal-resistant bacteria. Mercury- and tellurite-resistant strains were isolated from Greenwich Island (Rodriguez-Rojas et al., 2016b). *Pseudomonas putida* strain ATH-43 (Figure 2 and Table 2) revealed high tolerance to cadmium, copper, chromium, and selenium, whereas *Psychrobacter* sp. ATH-62 is resistant to mercury and tellurite (Rodriguez-Rojas et al., 2016a). Heavy metal-resistant bacteria were also isolated from invertebrates from the Antarctic shores. Mercury-resistant *Pseudoalteromonas* sp. gw196 and *Colwellia* sp. gw172, and zinc and cadmium-resistant *Arthrobacter* and *Psychrobacter* strains were isolated from a marine sponge (Mangano et al., 2013). *Flavobacterium* and *Psychrobacter* strains with resistance to mercury and zinc were isolated from a sea urchin (Gonzalez-Aravena et al., 2016).

Antarctic bacteria have been also studied for additional biotechnological applications. An Antarctic psychrophilic *Pseudomonas* strain was able to produce quantum dots from Cd salts (Gallardo et al., 2014). A psychrophilic *Pseudomonas mandelli* able to produce alginate has also been reported (Bayat et al., 2015; Vasquez-Ponce et al., 2017). Alginate is useful to encapsulate microbes to improve the bioremediation of persistent organic pollutants such as pesticides in polluted soils (Morgante et al., 2010). Encapsulation of microbes in alginate beads may prevent stress or predation of the inocula (Morgante et al., 2010).

The diversity of bacteria from the Antarctica that showed wide range heavy metal resistance and hydrocarbon degradation capabilities revealed a complex community that include adapted cosmopolitan bacteria such as *Pseudomonas* and other native strains, which may be useful for bioremediation in cold environments.

## Concluding Remarks and Future Perspectives

Chile has been referred to be a “biogeographic island” due to a succession of natural barriers, including Altiplano, Atacama Desert, Los Andes Mountains, South Pacific Ocean, Patagonia, and Antarctica. Geological processes and climatic conditions have driven the current geography of Chile into a set of diverse ecosystems that reassemble heaven for both extremophilic and polyextremophilic microorganisms. Those biotopes include a multiplicity of ecosystems with more than one extreme environmental condition, such a high salinity, low humidity, high UV radiation, high or low temperature, high or low pH, and high concentration of heavy metals. These extreme environmental conditions exert a strong pressure limiting the biodiversity of each niche, since every condition is associated with its own set of adaptations for stability on its environment. At specie-level, microbes that are capable to persist in such (multi) extreme conditions gather (multiple) metabolic capabilities that are not present in mesophilic microbes, and thus, represent a great potential for biotechnological and environmental applications (Liu et al., 2018). At an ecological level, it has been previously shown that microbial communities in extreme environments evolved at a faster rate than mesophiles living in benign environments, highlighting particularities of the evolutionary dynamic of such environments (Li et al., 2014). For these reasons, diverse research efforts have been initially focused on analyzing natural microbial communities of those sites and studying physiological and genomic characteristics of the microbial isolates.

Next generation sequencing (NGS) and next generation proteomics (NGPs) provide powerful techniques to gain insights about the molecular mechanisms involved in microbial adaptation to extreme conditions (Armengaud, 2016). These type of studies and methods will reveal the mechanisms and strategies used by microbes to adapt to extreme conditions and are useful to understand the evolution of microorganisms subjected to extreme conditions.

In recent years, there are significant advances in the field of extremophiles at the global level. Similar trend has also been reported by researchers from Chile, whose discoveries have made the field to flourish. This progress relied upon the advances of physico-chemical characterization of those biotopes together with the advent of high-throughput molecular tools to understand the complexity of microbial communities and improved tools and techniques for microbial isolation. Many of those studies have gained significant insights on the molecular mechanisms involved in extremophiles’ adaptations to extreme environments and their co-evolutionary processes, and also contributed to current biotechnological developments, such as Swissaustral, a company with headquarters in Chile, which performs functional enzymatic screening for industrial and biotechnological applications (Blamey et al., 2017). Additional research efforts must be invested into broadening the taxonomic breadth of extremophiles and expanding towards the study of extremolyes, metabolites utilized as biological defense mechanisms to combat extreme environmental stresses (such as ectoine).

The restoration of polluted environment is a crucial task for sustainable development. For the clean up of polluted sites, bioremediation is an attractive alternative to physico-chemical treatment processes. Microbes are main catalysts for the bioremediation of polluted environments. Extremophiles has been isolated in diverse zones in Chile that possess extreme conditions such as Altiplano, Atacama Desert, Central Chile, Patagonia, and Antarctica. Interestingly, the remarkable adaptative capabilities of extremophiles convert these organisms into an attractive source of catalysts for bioremediation and industrial processes.

Future research will carry out more detailed studies on extremophiles already described in order to deepen their knowledge and optimize their use in industrial processes including bioremediation. On the other side, a large number of extreme environments in Chile are still unexplored or underexplored. These habitats harbor microbial communities with metabolic capabilities useful for bioremediation processes and other applications. Therefore, future research should focus on the isolation, identification and characterization of a higher number of extremophiles from ecosystems with extreme conditions. Thereafter, studies should explore the metabolic potential of microbial isolates for application in biotechnology including the bioremediation of heavy metals and persistent organic pollutants. The basic research on extremophiles combined with the scale up of the biotechnological process including bioremediation and waste treatments will offer a bright future to Chilean economy and an increasing welfare to its inhabitants.

## Dedication

This article is dedicated to the memory of Professor Dr. Burkhard Seeger Stein (1929–2016), who inspired the study of natural resources in Chile through his pioneering research and teaching.

## Author Contributions

RO, CM, GB, FD, AC, RV, CR, and MS wrote and discussed the article.

## Conflict of Interest Statement

The authors declare that the research was conducted in the absence of any commercial or financial relationships that could be construed as a potential conflict of interest.
